# Non-invasive diagnostic criteria of hepatocellular carcinoma: Comparison of diagnostic accuracy of updated LI-RADS with clinical practice guidelines of OPTN-UNOS, AASLD, NCCN, EASL-EORTC, and KLSCG-NCC

**DOI:** 10.1371/journal.pone.0226291

**Published:** 2019-12-10

**Authors:** Burcu Erkan, Jeffrey Meier, Toshimasa J. Clark, Jeffrey Kaplan, Jeffrey R. Lambert, Samuel Chang

**Affiliations:** 1 Department of Radiology, University of Colorado School of Medicine, Aurora, Colorado, United States of America; 2 Department of Pathology, University of Colorado School of Medicine, Aurora, Colorado, United States of America; 3 Department of Biostatistics and Informatics, University of Colorado School of Public Health, Aurora, Colorado, United States of America; Centre de Recherche en Cancerologie de Lyon, FRANCE

## Abstract

**Purpose:**

To retrospectively compare the diagnostic performance of different noninvasive diagnostic criteria of HCC including LI-RADS, OPTN-UNOS, AASLD, NCCN, EASL-EORTC, KLCSG-NCC.

**Materials and methods:**

We reviewed the medical records of 3,491 pathologically examined liver lesions from January-2011 to January-2015 in our institution. 195 lesions in 133 patients (M:F = 100:33) with chronic hepatitis B/C and/or cirrhosis for any etiology were finally included in our study, with 98 lesions ≥ 2 cm, 72 lesions between 1–2 cm, and 25 lesions < 1 cm. The main comparison was made with the largest nodules of each patient (*n* = 133). The lesions were retrospectively evaluated for the diagnosis of HCC on DCE-CT or MR using different noninvasive diagnostic criteria including LI-RADS, OPTN-UNOS, AASLD, NCCN, EASL-EORTC, and KLCSG-NCC. With pathological evaluation serving as a gold-standard, sensitivity, specificity, PPV and NPV as well as accuracy of the diagnostic criteria were calculated.

**Results:**

There was no statistically significant differences in diagnostic accuracy among noninvasive diagnostic criteria. For 133 lesions of the largest lesion per patient, the overall accuracy was highest with LI-RADS criteria (89.3%) and the overall sensitivity was highest with LI-RADS, AASLD, NCCN criteria (all 89.5%). For 1–2 cm lesions, sensitivity decreased for all criteria in the following order: EASL-EORTC (59.1%), KLCSG-NCC (58.3%), LI-RADS, AASLD, NCCN (all 56.5%), and OPTN-UNOS (22.7%) criteria. OPTN-UNOS had the highest specificity in cirrhotic livers, 91.7%.

**Conclusions:**

The current noninvasive diagnostic criteria of HCC have no statistically significant difference in diagnostic accuracy. Overall, LI-RADS had the highest sensitivity and accuracy among the guidelines. OPTN had the highest specificity for cirrhotic livers.

## Introduction

Hepatocellular carcinoma (HCC) imposes a significant health problem in the world. The incidence of HCC is increasing worldwide and it’s amongst the leading causes of cancer-related death globally [1]. HCC can be diagnosed noninvasively when it demonstrates classical imaging features. Multiple international organizations have developed noninvasive diagnostic criteria. Except one, all others have been updated in 2018.

Among various guidelines, Liver Imaging Reporting and Data System (LI-RADS) has set the criteria using the standardized terminology to interpret and report imaging examinations of the liver in patients with cirrhosis or otherwise at risk for HCC [2]. The American College of Radiology (ACR) supported and endorsed LI-RADS, which was last revised in 2018 [3]. Organ Procurement and Transplantation Network (OPTN)-United Network for Organ Sharing (UNOS) (2018) (https://optn.transplant.hrsa.gov/media/1200/optn_policies.pdf) which determines the priority for organ distribution among patients has adopted LI-RADS for noninvasive diagnosis of HCC with minor modifications. They are the most commonly used noninvasive diagnostic criteria of HCC in the United States. LI-RADS is more popular among radiologists, whereas OPTN-UNOS criteria is more widely accepted among transplant surgeons and hepatologists. American Association for the Study of Liver Diseases (AASLD) (2018) [4] has recently adopted LI-RADS, OPTN-UNOS and the previous AASLD guideline (2011) [5] and National Comprehensive Cancer Network (NCCN) (2018) (https://www.nccn.org/professionals/physician_gls/PDF/hepatobiliary.pdf) has recently adopted AASLD (2011), European Association for the Study of the Liver—European Organization for Research and Treatment of Cancer (EASL-EORTC) (2012), LI-RADS (2014) and OPTN-UNOS (2010) for the imaging criteria on their latest update. With the integration of LI-RADS into the AASLD 2018 HCC clinical practice guideline, LI-RADS released an expedited update for the unification. EASL-EORTC criteria [1] which was also recently updated (2018), is the most commonly used criteria in Europe. Korean Liver Cancer Study Group (KLCSG)-National Cancer Center (NCC) (2014) [6], is the selected guideline for this study from Asia.

The purpose of this study is to estimate and compare the diagnostic accuracy of these multiple different noninvasive diagnostic criteria of HCC. The potential strengths and weaknesses of the different systems were also evaluated.

## Materials and methods

The Colorado Multiple Institutional Review Board (COMIRB) approved this retrospective study and informed consent was waived (COMIRB 15–0227).

### Subjects and lesions

Patients who had undergone liver explantation, resection or biopsy for focal hepatic lesions at our institution from January 2011 to January 2015 were reviewed. Patients with high risk factors of HCC including hepatitis B virus (HBV) infection, hepatitis C virus (HCV) infection, and/or cirrhosis for any etiology who fulfilled required examinations for diagnosis of HCC such as dynamic contrast-enhanced (DCE) Computed Tomography (CT) and/or Magnetic Resonance (MR) Imaging and had pathologic diagnosis of liver lesion within 1 year from imaging were included in this study. The presence of chronic liver disease or cirrhosis was determined based on any of clinician’s medical records, imaging findings, or pathological reports describing fibrosis stage 4 [7]. The patients treated prior to imaging were excluded, for treatment could change the imaging findings. The patients with totally necrotic lesions on pathology were excluded. The subjects younger than 18 years old and older than 89 years old were not included in the study for the ethical requirements.

The age, gender, viral markers of HBV, HCV, serum alpha-fetoprotein (AFP) level, clinical diagnosis of underlying liver disease, pathological results of liver lesion and the background liver parenchyma were collected from medical records of the subjects.

### Imaging and pathology

The CT, MR and ultrasound images were obtained from the picture archiving and communication system (PACS) of the institution. The minimum technical specifications of the imaging were DCE-CT (minimum three phases) with iodine-based contrast agent by multidetector CT scanner and/or DCE-MR (pre-contrast and minimum three post-contrast phases) with Gadolinium-based extracellular contrast media by 1.5 or 3 Tesla MR scanner. The essential phases for imaging were late-arterial, portal-venous and equilibrium phases. Late arterial phase was obtained approximately 14–16 seconds after triggering over 100 HU at the suprarenal abdominal aorta on CT and 8–10 seconds after the appearance of the contrast agent in the abdominal aorta on MR, by use of a bolus tracking techniques on both modalities. The enhancement of the hepatic vasculature was evaluated to determine adequate late arterial phase images. No further classifications were made between DCE-CT and DCE-MR, for they are considered equivalent by all noninvasive diagnostic criteria.

DCE-CT and/or MR prior to the invasive procedures were retrospectively evaluated, by three board-certified abdominal radiologists. When there are multiple examinations prior to treatment or procedures meeting the inclusion criteria, the examination closest to the treatment or procedure was reviewed. Blinded to pathology results and clinical radiologists’ reports and their interpretation, the imaging findings of the liver and the focal lesions such as; presence or absence of cirrhosis or morphologic findings suggestive of chronic liver disease, the diameter of liver lesions, arterial phase hyperenhancement (APHE), portal-venous phase or delayed (transitional for gadoxetic acid) phase washout, and capsule appearance were evaluated. The hepatobiliary phase findings of MR, new lesion on ultrasonography, threshold growth of the lesion, and macrovascular invasion were noted if available. The imaging findings for each of these were determined by the consensus among the three radiologists. Then, multiple different noninvasive diagnostic criteria of these guidelines of HCC (version 2018 for all, except KLCSG-NCC version 2014) were applied to these imaging findings of focal liver lesions, which categorized lesions into definitely HCC (LR-5) versus not definitely HCC (includes probably HCC, indeterminate probability of malignancy, probably benign, and definitely benign observations) for LI-RADS and definitely HCC versus indeterminate lesion for the other diagnostic criteria. The major differences of these noninvasive diagnostic criteria of HCC are summarized in Tables 1 & 2.

**Table 1:** Major difference between guidelines.

Guideline	Applicable patient group	Initiated modality	Minimum specifications of CT/MR	Nodule size range
LI-RADS	Cirrhosis* HBV Current or prior HCC	US surveillance CEUS diagnosis CT/MR diagnosis, staging	CT: Multidetector ≥8 rows, 3 phase required, precontrast suggested; MR: 1.5T or 3T, With extracellular contrast, Gadobenate dimeglumine, 4 phase (including precontrast), With Gadoxetate disodium, 5 phase (including HBP)	10-19mm ≥20mm
OPTN-UNOS	Cirrhosis	CT/MR	Must be interpreted by a radiologist at a transplant hospital, DCE CT/MR	10-19mm ≥20mm
AASLD	Cirrhosis HBV	US or US+AFP surveillance CT/MR diagnosis	Multiphasic CT/MR as LI-RADS	>10mm
NCCN	Cirrhosis HBV Current or prior HCC	US/AFP surveillance CT/MR diagnosis	Multiphasic CT with extracellular agents; Multiphasic MR with extracellular agents or with Gadoxetate disodium	>10mm
EASL-EORTC	Cirrhosis	US surveillance CT/MR diagnosis CEUS diagnosis	Multiphasic CT, DCE MR, Gadoxetic acid enhanced MR	>10mm
KLCSG-NCC	Cirrhosis HBV HCV	US/AFP surveillance CT/MR, AFP diagnosis	CT: Multidetector ≥4 rows; MR: 1.5T or 3T, With Gadolinium based extracellular contrast media, 4 phase (including precontrast), With Gadoxetate disodium, 5 phase (including HBP)	<10mm ≥10mm

*Do not apply cirrhosis due to congenital hepatic fibrosis, vascular disorders, <18 years old

CEUS: Contrast enhanced ultrasound

DCE: Dynamic contrast enhanced

HBP: Hepatobiliary phase

**Table 2:** Diagnostic criteria of the guidelines.

Nodule diameter	Criteria	LI-RADS	OPTN-UNOS	EASL-EORTC	AASLD NCCN	KLCSG-NCC
<10mm	Gradual increase of AFP on ≥2 consecutive exam and AH+WO on ≥2 exams (dynamic CT, MR, Gd-EOB-DTPA MR)	NA	NA	NA	NA	HCC
10-19mm	AH+WO AH+TG AH+WO+C AH+WO+TG AH+C+TG AH+WO+C+TG	LR-5 LR-5 LR-5 LR-5 LR-5 LR-5	- 5A-g 5A 5A - 5A	HCC - (biopsy) HCC HCC - (biopsy) HCC	HCC HCC HCC HCC HCC HCC	HCC - (Biopsy or follow up) HCC HCC - (Biopsy or follow up) HCC
≥20mm	AH+WO AH+C AH+TG AH+WO+C AH+WO+TG AH+C+TG AH+WO+C+TG	LR-5 LR-5 LR-5 LR-5 LR-5 LR-5 LR-5	5B 5B 5B 5B 5B 5B 5B	HCC - (biopsy) - (biopsy) HCC HCC - (biopsy) HCC	HCC HCC HCC HCC HCC HCC HCC	HCC - (Biopsy or follow up) - (Biopsy or follow up) HCC HCC - (Biopsy or follow up) HCC

NA: Not applicable

LR-5: Definitely HCC

AH: Arterial hyperenhancement

WO: Washout

C: Capsule appearance

TG: Threshold growth; ≤6 months ≥50% diameter increase

Pathology reports were reviewed. Pathological assessment was made after liver explantation for orthotopic liver transplantation (*n* = 110), tumor resection (*n* = 13) or by biopsy alone (*n* = 10). The pathologic specimens were sliced and examined in 5 mm thickness per-routine protocol for liver specimen exam of the pathology department of our institution. If there were any uncertainty in pathology reports, a liver pathologist reviewed the slides to confirm the diagnosis.

The results of imaging evaluation were compared to the gold standard, pathology reports, and analyzed statistically.

### Data analysis

Since guidelines have various criteria to define HCC depending on the size of the lesion, further sub-classifications were made for lesions ≥2cm and between 1-2cm. Except of KLCSG-NCC, no other classification categorizes lesions <1cm as HCC, therefore no comparison was made in such lesions.

Performance of the diagnostic criteria compared to pathology was evaluated using the group of one largest lesion per patient. Sensitivities, specificities, accuracies, PPV and negative predictive values (NPV) are reported for each diagnostic criteria. In addition, sub-analyses were performed for the groups of the largest lesion per patient ≥2 cm, the largest lesion per patient between 1–2 cm, the largest lesion per patient with liver cirrhosis ≥1cm, the largest lesion per patient with cirrhosis ≥2 cm, and the largest lesion per patient with cirrhosis between 1–2 cm.

The degree of disagreement between the 6 diagnostic methods was assessed where applicable. If there was no disagreement between two methods, a significance test was not performed. Where a significance test was applicable, the 2-sided mid-*p* test presented by Fagerland et al (2013) was used as an alternative to the McNemar test for paired binary data, due to the small number of discordant pairs between the various methodologies. To protect our type I error rate against multiple hypothesis testing, we used a Bonferonni correction to adjust our level of statistical significance.

Tests were performed in both cases and controls as defined by pathology. The null hypothesis for the significance tests states that there is no difference in the marginal probabilities of both tests. In cases, this would indicate no difference in sensitivities and similarly, in controls this would indicate no difference in specificities.

## Results and discussion

**Fig 1:**
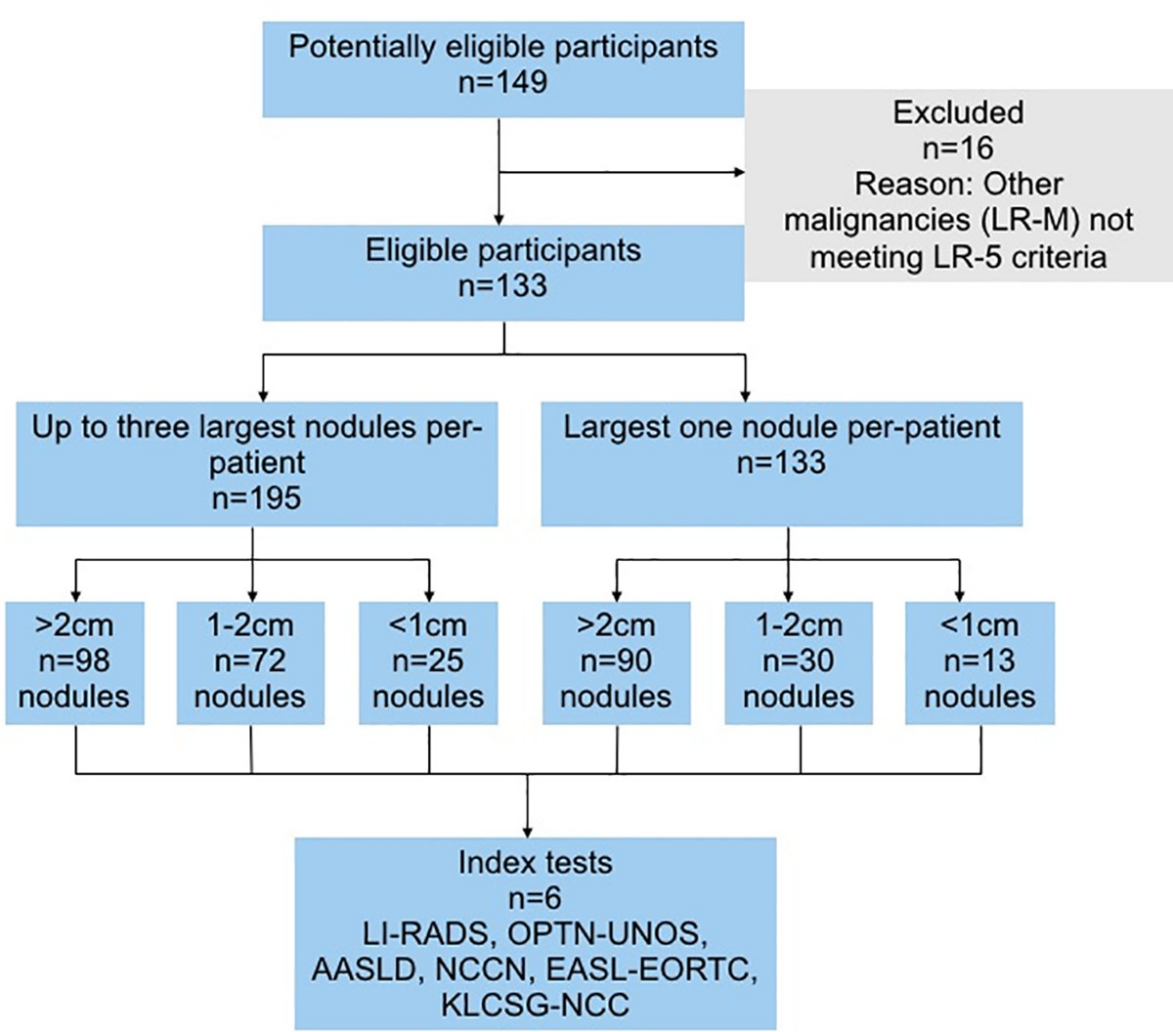
The diagram of the flow of the participants through the study.

**Fig 2:**
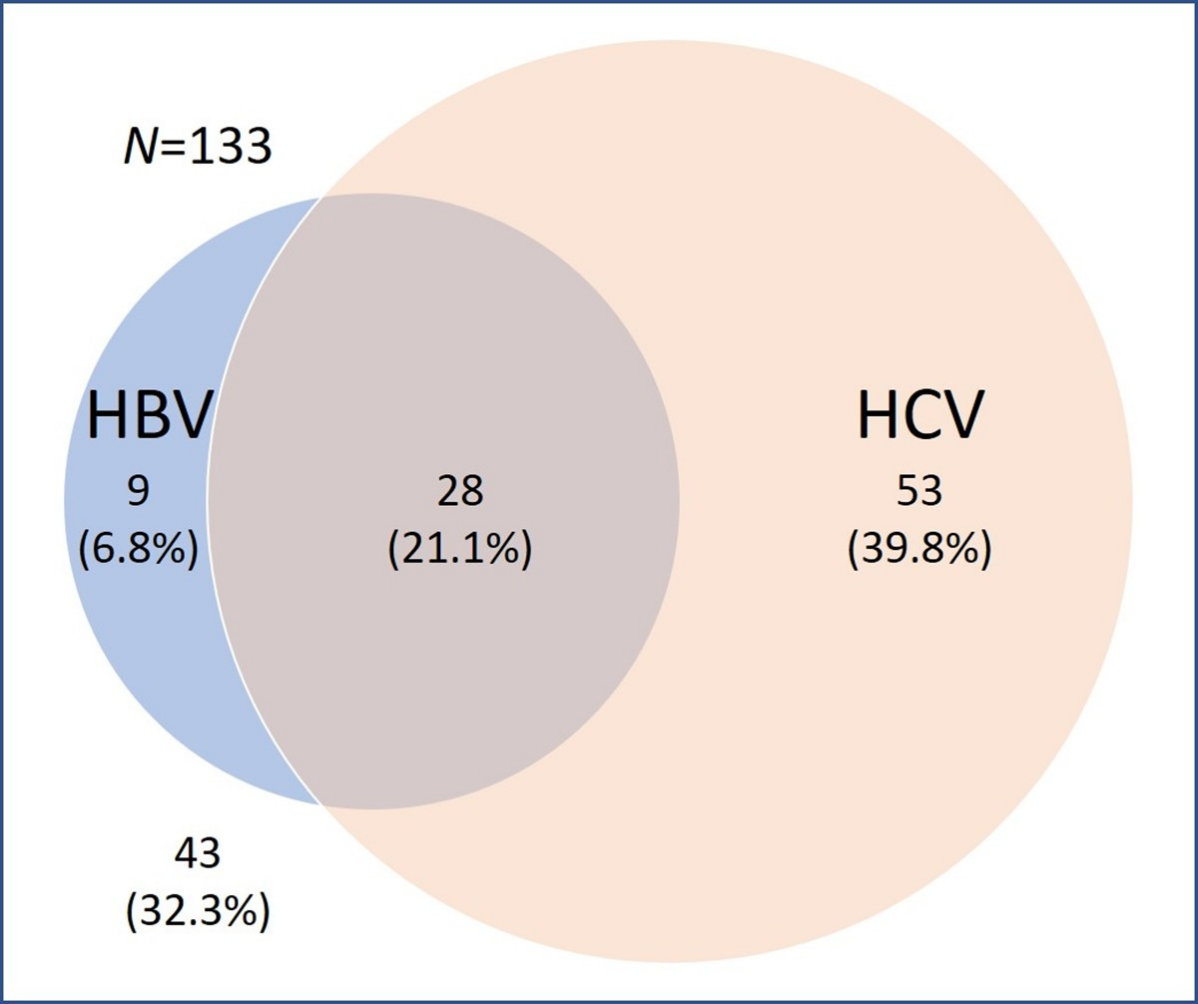
Distribution of the viral markers.

We reviewed the medical records of 3,491 patients who have undergone biopsy, resection, or explantation and subsequent pathological examination of the liver. Among these patients, 149 patients had both CT or MR reports and pathology reports of the focal hepatic lesions within 1 year. Sixteen patients were excluded due to the presence of obvious radiological findings of other malignancies. The flow of the participants through the study is depicted in Fig 1. Finally, 133 patients (M:F = 100:33, median age 58 years, range 30–87 years) with chronic hepatitis B/C and/or cirrhosis for any etiology who had focal hepatic lesions ≥ 5 mm reported on DCE-CT (*n* = 82) or MR (*n* = 51) were included. One hundred and twenty one patients had cirrhosis that was proven by pathology (*n* = 118) or by imaging (*n* = 3). Twelve patients were without cirrhosis despite HBV (*n* = 4) and HCV (*n* = 8) infection. The distribution of the viral markers is shown in Fig 2. AFP levels were available in 103 patients with a median value of 7.5 ng/ml (1.6 to 5,574.5 ng/ml) and mean value of 151.8 ng/ml.

Up to the three largest lesions were evaluated per patient. The total number of evaluated lesions was 195 with 98 lesions ≥ 2cm, 72 lesions between 1-2cm, and 25 lesions < 1cm. On the measurement of the largest diameter of the largest one lesion per patient, 90 lesions were ≥ 2cm, 30 lesions were between 1-2cm, and 13 lesions were < 1cm. The median interval between pathological assessment and imaging was 111 days and the mean interval was 139 days. In cirrhotic livers, there were 108 lesions ≥ 1cm and 80 lesions ≥ 2cm, to which OPTN-UNOS and EASL-EORTC criteria were applied. One hundred and twelve lesions were ≥ 1cm and 83 lesions were ≥ 2cm in cirrhosis and/or HBV patients, to which LIRADS, AASLD, and NCCN criteria were applied.

Pathologically a total of 112 lesions were diagnosed as HCC. The rest were diagnosed as cholangiocarcinoma (*n* = 1), angiomyolipoma (*n* = 1), hemangioma (*n* = 1), benign vascular lesion (*n* = 1), arterio-portal shunt (*n* = 1), macro regenerative nodules (*n* = 9), dysplastic nodule (*n* = 6), necrosis (*n* = 2), and other (cirrhotic liver changes or nontumoral, *n* = 61). The supporting information of the nodule diameters, underlying conditions (HBV, HCV, cirrhosis), pathology and consensus results for different diagnostic criteria can be found on S1 Table.

There was no statistically significant differences in diagnostic performance between noninvasive diagnostic criteria. The overall accuracy was highest with LI-RADS criteria for all analysis. For the group of the largest lesion per patient of all size, the accuracy was highest with LI-RADS (89.3%), followed by AASLD, NCCN (all 88.4%), KLCSG-NCC (87.2%), EASL-EORTC (87%) and OPTN-UNOS (85.2%) criteria. For the largest lesions per patient ≥2 cm, the accuracy was equal in LI-RADS, AASLD and NCCN criteria (88%), followed by OPTN-UNOS (87.5%), KLCSG-NCC (86.7%) and EASL-EORTC (85%). The accuracy decreases for all criteria for 1–2 cm lesions that was highest with LI-RADS (79.6%), followed by EASL-EORTC (79.2%), KLCSG-NCC (78.2%), AASLD, NCCN (all 77.8%), and lowest with OPTN-UNOS (67.9%) criteria.

For the group of the largest lesion per patient of all size, the sensitivity was highest with LI-RADS, AASLD, NCCN criteria (all 89.5%). For 1–2 cm lesions sensitivities were decreased for all criteria with the highest being EASL (59.1%) followed by KLCSG (58.3%), LI-RADS, AASLD, NCCN (all 56.5%) and lowest with OPTN (22.7%) criteria. The specificity was highest with OPTN-UNOS (91.7%) criteria followed by LI-RADS (88.9%), KLCSG-NCC (89.6%) and AASLD, NCCN, EASL-EORTC (all 86.1%).

The sub-analyses for cirrhosis patients limited the study population eligible for each guideline. For the group of the largest lesion per patient with cirrhotic liver, LI-RADS had the highest accuracy (89.9%). OPTN-UNOS had the highest specificity (91.7%) despite its relatively low sensitivity (81.9%) in cirrhotic patients. Table 3 show the specificities, sensitivities, NPV, PPV, and accuracies of the six different guidelines.

**Table 3:** Diagnostic performance of noninvasive diagnostic criteria.

**Largest lesion per patient, all size**						
**Criteria**	***N***	**Specificity**	**Sensitivity**	**NPV**	**PPV**	**Accuracy**
LI-RADS	112	0.889 (0.739, 0.969)	0.895 (0.803, 0.953)	0.800 (0.644, 0.909)	0.944 (0.864, 0.985)	0.893 (0.820, 0.943)
OPTN	108	0.917 (0.775, 0.982)	0.819 (0.711, 0.900)	0.717 (0.565, 0.840)	0.952 (0.865, 0.990)	0.852 (0.771, 0.913)
AASLD	112	0.861 (0.705, 0.953)	0.895 (0.803, 0.953)	0.795 (0.635, 0.907)	0.932 (0.847, 0.977)	0.884 (0.810, 0.937)
NCCN	112	0.861 (0.705, 0.953)	0.895 (0.803, 0.953)	0.795 (0.635, 0.907)	0.932 (0.847, 0.977)	0.884 (0.810, 0.937)
EASL	108	0.861 (0.705, 0.953)	0.875 (0.776, 0.941)	0.775 (0.615, 0.892)	0.926 (0.837, 0.976)	0.870 (0.792, 0.927)
KLCSG	133	0.896 (0.773, 0.965)	0.859 (0.766, 0.925)	0.782 (0.650, 0.882)	0.936 (0.857, 0.979)	0.872 (0.803, 0.924)
**Largest lesion per patient ≥2cm**						
LI-RADS	83	0.812 (0.544, 0.960)	0.896 (0.797, 0.957)	0.650 (0.408, 0.846)	0.952 (0.867, 0.990)	0.880 (0.790, 0.941)
OPTN	80	0.812 (0.544, 0.960)	0.891 (0.788, 0.955)	0.650 (0.408, 0.846)	0.950 (0.861, 0.990)	0.875 (0.782, 0.938)
AASLD	83	0.812 (0.544, 0.960)	0.896 (0.797, 0.957)	0.650 (0.408, 0.846)	0.952 (0.867, 0.990)	0.880 (0.790, 0.941)
NCCN	83	0.812 (0.544, 0.960)	0.896 (0.797, 0.957)	0.650 (0.408, 0.846)	0.952 (0.867, 0.99)	0.880 (0.790, 0.941)
EASL	80	0.812 (0.544, 0.960)	0.859 (0.750, 0.934)	0.591 (0.364, 0.793)	0.948 (0.856, 0.989)	0.850 (0.753, 0.920)
KLCSG	90	0.824 (0.566, 0.962)	0.877 (0.779, 0.942)	0.609 (0.385, 0.803)	0.955 (0.875, 0.991)	0.867 (0.779, 0.929)
**Largest lesion per patient between 1cm and 2cm**						
LI-RADS	29	0.968 (0.833, 0.999)	0.565 (0.345, 0.768)	0.750 (0.588, 0.873)	0.929 (0.661, 0.998)	0.796 (0.665, 0.894)
OPTN	28	1.000 (0.888, 1.000)	0.227 (0.078, 0.454)	0.646 (0.495, 0.778)	1.000 (0.478, 1.000)	0.679 (0.537, 0.801)
AASLD	29	0.935 (0.786, 0.992)	0.565 (0.345, 0.768)	0.744 (0.579, 0.870)	0.867 (0.595, 0.983)	0.778 (0.644, 0.880)
NCCN	29	0.935 (0.786, 0.992)	0.565 (0.345, 0.768)	0.744 (0.579, 0.870)	0.867 (0.595, 0.983)	0.778 (0.644, 0.880)
EASL	28	0.935 (0.786, 0.992)	0.591 (0.364, 0.793)	0.763 (0.598, 0.886)	0.867 (0.595, 0.983)	0.792 (0.659, 0.892)
KLCSG	30	0.935 (0.786, 0.992)	0.583 (0.366, 0.779)	0.744 (0.579, 0.870)	0.875 (0.617, 0.984)	0.782 (0.650, 0.882)
**Largest lesion per patient ≥1cm in patients with cirrhosis**						
LI-RADS	108	0.889 (0.739, 0.969)	0.903 (0.810, 0.960)	0.821 (0.665, 0.925)	0.942 (0.858, 0.984)	0.898 (0.825, 0.948)
OPTN	108	0.917 (0.775, 0.982)	0.819 (0.711, 0.900)	0.717 (0.565, 0.840)	0.952 (0.865, 0.990)	0.852 (0.771, 0.913)
AASLD	108	0.861 (0.705, 0.953)	0.903 (0.810, 0.960)	0.816 (0.657, 0.923)	0.929 (0.841, 0.976)	0.889 (0.814, 0.941)
NCCN	108	0.861 (0.705, 0.953)	0.903 (0.810, 0.960)	0.816 (0.657, 0.923)	0.929 (0.841, 0.976)	0.889 (0.814, 0.941)
EASL	108	0.861 (0.705, 0.953)	0.875 (0.776, 0.941)	0.775 (0.615, 0.892)	0.926 (0.837, 0.976)	0.870 (0.792, 0.927)
KLCSG	108	0.861 (0.705, 0.953)	0.875 (0.776, 0.941)	0.775 (0.615, 0.892)	0.926 (0.837, 0.976)	0.870 (0.792, 0.927)
**Largest lesion per patient ≥2cm in patients with cirrhosis**						
LI-RADS	80	0.812 (0.544, 0.960)	0.891 (0.788, 0.955)	0.650 (0.408, 0.846)	0.950 (0.861, 0.990)	0.875 (0.782, 0.938)
OPTN	80	0.812 (0.544, 0.960)	0.891 (0.788, 0.955)	0.650 (0.408, 0.846)	0.950 (0.861, 0.990)	0.875 (0.782, 0.938)
AASLD	80	0.812 (0.544, 0.960)	0.891 (0.788, 0.955)	0.650 (0.408, 0.846)	0.950 (0.861, 0.990)	0.875 (0.782, 0.938)
NCCN	80	0.812 (0.544, 0.960)	0.891 (0.788, 0.955)	0.650 (0.408, 0.846)	0.950 (0.861, 0.990)	0.875 (0.782, 0.938)
EASL	80	0.812 (0.544, 0.960)	0.859 (0.750, 0.934)	0.591 (0.364, 0.793)	0.948 (0.856, 0.989)	0.850 (0.753, 0.920)
KLCSG	80	0.812 (0.544, 0.960)	0.859 (0.750, 0.934)	0.591 (0.364, 0.793)	0.948 (0.856, 0.989)	0.850 (0.753, 0.920)
**Largest lesion per patient between 1cm and 2cm in patients with cirrhosis**						
LI-RADS	28	0.968 (0.833, 0.999)	0.591 (0.364, 0.793)	0.769 (0.607, 0.889)	0.929 (0.661, 0.998)	0.811 (0.680, 0.906)
OPTN	28	1.000 (0.888, 1.000)	0.227 (0.078, 0.454)	0.646 (0.495, 0.778)	1.000 (0.478, 1.000)	0.679 (0.537, 0.801)
AASLD	28	0.935 (0.786, 0.992)	0.591 (0.364, 0.793)	0.763 (0.598, 0.886)	0.867 (0.595, 0.983)	0.792 (0.659, 0.892)
NCCN	28	0.935 (0.786, 0.992)	0.591 (0.364, 0.793)	0.763 (0.598, 0.886)	0.867 (0.595, 0.983)	0.792 (0.659, 0.892)
EASL	28	0.935 (0.786, 0.992)	0.591 (0.364, 0.793)	0.763 (0.598, 0.886)	0.867 (0.595, 0.983)	0.792 (0.659, 0.892)
KLCSG	28	0.935 (0.786, 0.992)	0.591 (0.364, 0.793)	0.763 (0.598, 0.886)	0.867 (0.595, 0.983)	0.792 (0.659, 0.892)

*N*: Number of lesions evaluated by each noninvasive diagnostic criterion

There was some discordance in total number of HCC lesions due to differences in eligibility requirements of different criteria to define a lesion as HCC. These differences are summarized in Table 4. Sixteen separate hypothesis tests were performed comparing the various methodologies. The Bonferonni corrected level of significance is therefore 0.05/16 = 0.003125, and this value was used to assess statistical significance. Taken together, these discordances (Table 5) did not create any statistically significant differences.

**Table 4:** Comparison of tests, discordant tables.

**Discordant pairs (cases)**						
**Largest nodule per patient**						
**Criteria**	**LIRADS**	**OPTN**	**AASLD**	**NCCN**	**EASL**	**KLCSG**
LIRADS	NA	6(8.33%)	0(0%)	0(0%)	2(2.78%)	2(2.63%)
OPTN	6(8.33%)	NA	6(8.33%)	6(8.33%)	8(11.11%)	8(11.11%)
AASLD	0(0%)	6(8.33%)	NA	0(0%)	2(2.78%)	2(2.63%)
NCCN	0(0%)	6(8.33%)	0(0%)	NA	2(2.78%)	2(2.63%)
EASL	2(2.78%)	8(11.11%)	2(2.78%)	2(2.78%)	NA	0(0%)
KLCSG	2(2.63%)	8(11.11%)	2(2.63%)	2(2.63%)	0(0%)	NA
**Largest nodule ≥2cm**						
LIRADS	NA	0(0%)	0(0%)	0(0%)	2(3.12%)	2(2.99%)
OPTN	0(0%)	NA	0(0%)	0(0%)	2(3.12%)	2(3.12%)
AASLD	0(0%)	0(0%)	NA	0(0%)	2(3.12%)	2(2.99%)
NCCN	0(0%)	0(0%)	0(0%)	NA	2(3.12%)	2(2.99%)
EASL	2(3.12%)	2(3.12%)	2(3.12%)	2(3.12%)	NA	0(0%)
KLCSG	2(2.99%)	2(3.12%)	2(2.99%)	2(2.99%)	0(0%)	NA
**Largest nodule between 1cm and 2cm**						
LIRADS	NA	8(36.36%)	0(0%)	0(0%)	0(0%)	0(0%)
OPTN	8(36.36%)	NA	8(36.36%)	8(36.36%)	8(36.36%)	8(36.36%)
AASLD	0(0%)	8(36.36%)	NA	0(0%)	0(0%)	0(0%)
NCCN	0(0%)	8(36.36%)	0(0%)	NA	0(0%)	0(0%)
EASL	0(0%)	8(36.36%)	0(0%)	0(0%)	NA	0(0%)
KLCSG	0(0%)	8(36.36%)	0(0%)	0(0%)	0(0%)	NA
**Largest nodule in patients with cirrhosis**						
LIRADS	NA	6(8.33%)	0(0%)	0(0%)	2(2.78%)	2(2.78%)
OPTN	6(8.33%)	NA	6(8.33%)	6(8.33%)	8(11.11%)	8(11.11%)
AASLD	0(0%)	6(8.33%)	NA	0(0%)	2(2.78%)	2(2.78%)
NCCN	0(0%)	6(8.33%)	0(0%)	NA	2(2.78%)	2(2.78%)
EASL	2(2.78%)	8(11.11%)	2(2.78%)	2(2.78%)	NA	0(0%)
KLCSG	2(2.78%)	8(11.11%)	2(2.78%)	2(2.78%)	0(0%)	NA
**Largest nodule ≥2cm in patients with cirrhosis**						
LIRADS	NA	0(0%)	0(0%)	0(0%)	2(3.12%)	2(3.12%)
OPTN	0(0%)	NA	0(0%)	0(0%)	2(3.12%)	2(3.12%)
AASLD	0(0%)	0(0%)	NA	0(0%)	2(3.12%)	2(3.12%)
NCCN	0(0%)	0(0%)	0(0%)	NA	2(3.12%)	2(3.12%)
EASL	2(3.12%)	2(3.12%)	2(3.12%)	2(3.12%)	NA	0(0%)
KLCSG	2(3.12%)	2(3.12%)	2(3.12%)	2(3.12%)	0(0%)	NA
**Largest nodule between 1cm and 2cm in patients with cirrhosis**						
LIRADS	NA	8(36.36%)	0(0%)	0(0%)	0(0%)	0(0%)
OPTN	8(36.36%)	NA	8(36.36%)	8(36.36%)	8(36.36%)	8(36.36%)
AASLD	0(0%)	8(36.36%)	NA	0(0%)	0(0%)	0(0%)
NCCN	0(0%)	8(36.36%)	0(0%)	NA	0(0%)	0(0%)
EASL	0(0%)	8(36.36%)	0(0%)	0(0%)	NA	0(0%)
KLCSG	0(0%)	8(36.36%)	0(0%)	0(0%)	0(0%)	NA
**Discordant pairs (controls)**						
**Largest nodule per patient**						
**Criteria**	**LIRADS**	**OPTN**	**AASLD**	**NCCN**	**EASL**	**KLCSG**
LIRADS	NA	1(2.78%)	1(2.78%)	1(2.78%)	1(2.78%)	1(2.78%)
OPTN	1(2.78%)	NA	2(5.56%)	2(5.56%)	2(5.56%)	2(5.56%)
AASLD	1(2.78%)	2(5.56%)	NA	0(0%)	0(0%)	0(0%)
NCCN	1(2.78%)	2(5.56%)	0(0%)	NA	0(0%)	0(0%)
EASL	1(2.78%)	2(5.56%)	0(0%)	0(0%)	NA	0(0%)
KLCSG	1(2.78%)	2(5.56%)	0(0%)	0(0%)	0(0%)	NA
**Largest nodule ≥2cm**						
LIRADS	NA	0(0%)	0(0%)	0(0%)	0(0%)	0(0%)
OPTN	0(0%)	NA	0(0%)	0(0%)	0(0%)	0(0%)
AASLD	0(0%)	0(0%)	NA	0(0%)	0(0%)	0(0%)
NCCN	0(0%)	0(0%)	0(0%)	NA	0(0%)	0(0%)
EASL	0(0%)	0(0%)	0(0%)	0(0%)	NA	0(0%)
KLCSG	0(0%)	0(0%)	0(0%)	0(0%)	0(0%)	NA
**Largest nodule between 1cm and 2cm**						
LIRADS	NA	1(3.23%)	1(3.23%)	1(3.23%)	1(3.23%)	1(3.23%)
OPTN	1(3.23%)	NA	2(6.45%)	2(6.45%)	2(6.45%)	2(6.45%)
AASLD	1(3.23%)	2(6.45%)	NA	0(0%)	0(0%)	0(0%)
NCCN	1(3.23%)	2(6.45%)	0(0%)	NA	0(0%)	0(0%)
EASL	1(3.23%)	2(6.45%)	0(0%)	0(0%)	NA	0(0%)
KLCSG	1(3.23%)	2(6.45%)	0(0%)	0(0%)	0(0%)	NA
**Largest nodule in patients with cirrhosis**						
LIRADS	NA	1(2.78%)	1(2.78%)	1(2.78%)	1(2.78%)	1(2.78%)
OPTN	1(2.78%)	NA	2(5.56%)	2(5.56%)	2(5.56%)	2(5.56%)
AASLD	1(2.78%)	2(5.56%)	NA	0(0%)	0(0%)	0(0%)
NCCN	1(2.78%)	2(5.56%)	0(0%)	NA	0(0%)	0(0%)
EASL	1(2.78%)	2(5.56%)	0(0%)	0(0%)	NA	0(0%)
KLCSG	1(2.78%)	2(5.56%)	0(0%)	0(0%)	0(0%)	NA
**Largest nodule ≥2cm in patients with cirrhosis**						
LIRADS	NA	0(0%)	0(0%)	0(0%)	0(0%)	0(0%)
OPTN	0(0%)	NA	0(0%)	0(0%)	0(0%)	0(0%)
AASLD	0(0%)	0(0%)	NA	0(0%)	0(0%)	0(0%)
NCCN	0(0%)	0(0%)	0(0%)	NA	0(0%)	0(0%)
EASL	0(0%)	0(0%)	0(0%)	0(0%)	NA	0(0%)
KLCSG	0(0%)	0(0%)	0(0%)	0(0%)	0(0%)	NA
**Largest nodule between 1cm and 2cm in patients with cirrhosis**						
LIRADS	NA	1(3.23%)	1(3.23%)	1(3.23%)	1(3.23%)	1(3.23%)
OPTN	1(3.23%)	NA	2(6.45%)	2(6.45%)	2(6.45%)	2(6.45%)
AASLD	1(3.23%)	2(6.45%)	NA	0(0%)	0(0%)	0(0%)
NCCN	1(3.23%)	2(6.45%)	0(0%)	NA	0(0%)	0(0%)
EASL	1(3.23%)	2(6.45%)	0(0%)	0(0%)	NA	0(0%)
KLCSG	1(3.23%)	2(6.45%)	0(0%)	0(0%)	0(0%)	NA

Case: Nodule identified as cancerous based on pathology

Control: Nodule identified as being without cancer based on pathology

NA: Not applicable

**Table 5:** Discordance tests.

**Largest nodule per patient**						
**Test 1**	**Test 2**	***N***	**Number of disagreements**	**Test 2 incorrect**	**Test 1 incorrect**	**mid-*p***
LI-RADS	OPTN	108	7 (6.48%)	6	1	0.07031
LI-RADS	AASLD	112	1 (0.89%)	1	0	0.5
LI-RADS	NCCN	112	1 (0.89%)	1	0	0.5
LI-RADS	EASL	108	3 (2.78%)	3	0	0.125
LI-RADS	KLCSG	112	3 (2.68%)	3	0	0.125
OPTN	AASLD	108	8 (7.41%)	2	6	0.1797
OPTN	NCCN	108	8 (7.41%)	2	6	0.1797
OPTN	EASL	108	10 (9.26%)	4	6	0.5488
OPTN	KLCSG	108	10 (9.26%)	4	6	0.5488
AASLD	EASL	108	2 (1.85%)	2	0	0.25
AASLD	KLCSG	112	2 (1.79%)	2	0	0.25
NCCN	EASL	108	2 (1.85%)	2	0	0.25
NCCN	KLCSG	112	2 (1.79%)	2	0	0.25
**Largest nodule >2cm**						
LIRADS	EASL	80	2 (2.5%)	2	0	0.25
LIRADS	KLCSG	83	2 (2.41%)	2	0	0.25
OPTN	EASL	80	2 (2.5%)	2	0	0.25
OPTN	KLCSG	80	2 (2.5%)	2	0	0.25
AASLD	EASL	80	2 (2.5%)	2	0	0.25
AASLD	KLCSG	83	2 (2.41%)	2	0	0.25
NCCN	EASL	80	2 (2.5%)	2	0	0.25
NCCN	KLCSG	83	2 (2.41%)	2	0	0.25
**Largest nodule between 1 and 2cm**						
LIRADS	OPTN	28	9 (16.98%)	8	1	0.02148
LIRADS	AASLD	29	1 (1.85%)	1	0	0.5
LIRADS	NCCN	29	1 (1.85%)	1	0	0.5
LIRADS	EASL	28	1 (1.89%)	1	0	0.5
LIRADS	KLCSG	29	1 (1.85%)	1	0	0.5
OPTN	AASLD	28	10 (18.87%)	2	8	0.06543
OPTN	NCCN	28	10 (18.87%)	2	8	0.06543
OPTN	EASL	28	10 (18.87%)	2	8	0.06543
OPTN	KLCSG	28	10 (18.87%)	2	8	0.06543
**Largest nodule in patients with cirrhosis**						
LIRADS	OPTN	108	7 (6.48%)	6	1	0.07031
LIRADS	AASLD	108	1 (0.93%)	1	0	0.5
LIRADS	NCCN	108	1 (0.93%)	1	0	0.5
LIRADS	EASL	108	3 (2.78%)	3	0	0.125
LIRADS	KLCSG	108	3 (2.78%)	3	0	0.125
OPTN	AASLD	108	8 (7.41%)	2	6	0.1797
OPTN	NCCN	108	8 (7.41%)	2	6	0.1797
OPTN	EASL	108	10 (9.26%)	4	6	0.5488
OPTN	KLCSG	108	10 (9.26%)	4	6	0.5488
AASLD	EASL	108	2 (1.85%)	2	0	0.25
AASLD	KLCSG	108	2 (1.85%)	2	0	0.25
NCCN	EASL	108	2 (1.85%)	2	0	0.25
NCCN	KLCSG	108	2 (1.85%)	2	0	0.25
**Largest nodule > 2cm in patients with cirrhosis**						
LIRADS	EASL	80	2 (2.5%)	2	0	0.25
LIRADS	KLCSG	80	2 (2.5%)	2	0	0.25
OPTN	EASL	80	2 (2.5%)	2	0	0.25
OPTN	KLCSG	80	2 (2.5%)	2	0	0.25
AASLD	EASL	80	2 (2.5%)	2	0	0.25
AASLD	KLCSG	80	2 (2.5%)	2	0	0.25
NCCN	EASL	80	2 (2.5%)	2	0	0.25
NCCN	KLCSG	80	2 (2.5%)	2	0	0.25
**Largest nodule between 1 and 2cm in patients with cirrhosis**						
LIRADS	OPTN	28	9 (16.98%)	8	1	0.02148
LIRADS	AASLD	28	1 (1.89%)	1	0	0.5
LIRADS	NCCN	28	1 (1.89%)	1	0	0.5
LIRADS	EASL	28	1 (1.89%)	1	0	0.5
LIRADS	KLCSG	28	1 (1.89%)	1	0	0.5
OPTN	AASLD	28	10 (18.87%)	2	8	0.06543
OPTN	NCCN	28	10 (18.87%)	2	8	0.06543
OPTN	EASL	28	10 (18.87%)	2	8	0.06543
OPTN	KLCSG	28	10 (18.87%)	2	8	0.06543

*N*: Number of lesions

In summary, based on the consensus reading, none of the methodologies out-performs any of the other methodologies. This relationship is true in all of the subanalyses performed.

HCC is the most common primary liver cancer. It is the fifth most common cancer and the second most frequent cause of cancer-related deaths worldwide [8, 9]. HCC can be noninvasively diagnosed with relatively high accuracy using some combinations of imaging findings. The overall diagnostic accuracy increases in conjunction with the diameter of the lesion as the result of this study shows. The confirmative diagnosis of HCC is crucial for the decision of treatment strategy and it also eventually affects critical issues such as organ allocation. Therefore, many organizations including government authorities have developed the noninvasive diagnostic criteria of HCC. Furthermore, the diagnosis of HCC without invasive procedures decreases the risk of the procedure-related complications such as bleeding, infection, or tumor seeding as well as the costs [10, 11].

There are some differences between guidelines. According to the LI-RADS criteria, the clinical diagnosis of HCC without biopsy mainly relies on imaging criteria only, whereas elevated AFP level is not a major hallmark anymore on most of the guidelines. In the majority of guidelines the major hallmarks are APHE and portal-venous or delayed (transitional for gadoxetic acid) phase washout appearance of the lesion. LI-RADS includes more parameters in its structure such as capsule appearance, threshold growth, and major vascular invasion [11]. Unenhanced ultrasound for screening and surveillance and using contrast-enhanced ultrasound (CEUS) for diagnosis of HCC are the new diagnostic algorithms in cirrhotic and other high-risk patients that have launched in LI-RADS version 2017. On version 2018 LI-RADS made some revisions to the definition of the LI-RADS major feature, threshold growth, and to one LI-RADS category 5 criterion with the nodules between 10–19 mm in diameter.

EASL-EORTC (2018) added gadoxetic acid and CEUS on their latest update. Presence of a tumor capsule and significant tumor growth over time which is major finding for LI-RADS are not accepted by EASL, since they have not been prospectively validated [1]. Similarly in KLCSG-NCC guideline capsule appearance is considered as ancillary feature and do not increase the diagnostic accuracy beyond the dynamic criteria [12]. In KLCSG-NCC criteria (2014) tumor markers are one of the key factors in noninvasive diagnosis of HCC. The major difference of KLCSG-NCC from the other guidelines is the non-invasive diagnosis of lesions smaller than 1 cm in diameter and inclusion of treatment scheme. These lesions can be diagnosed non-invasively with the AFP level increase with the typical imaging findings. Also HCV infection is accepted as high risk besides the other factors for developing HCC in this guideline [6].

There are some studies of exploring the accuracy of the practical guidelines in literature, very few of which have done comparison between the guidelines [13–16]. However, the guidelines have been recently updated, and to our knowledge there is no published study comparing the latest versions of LI-RADS to other organizations’ practice guidelines. Our purpose was to see the effect of the differences in these noninvasive diagnostic criteria of HCC, when it's diagnosed with the multiple different guidelines. We have shown in this study that none of the criteria that we compare have a statistically significant superiority in HCC identification. The diagnostic performance including sensitivity, specificity, PPV, NPV, accuracies is not statistically significant. This result is seen both in the evaluation of all the lesions in all patient groups eligible for each criterion and in the subanalyses of the lesions in patients with cirrhosis only.

A study comparing the accuracy of AASLD and LI-RADS (version 2014) criteria for the non-invasive diagnosis of HCC smaller than 3 cm provided the information MR and CT separately [17]. They published no statistically significant difference for the sensitivity and specificity of AASLD and LI-RADS (combination of LR-5+LR5V) for entire cohort of lesions but lower sensitivity and higher specificity of LI-RADS than AASLD for lesions smaller than 20 mm by MR. For the entire cohort the sensitivity of the LI-RADS was not statistically different from that of the AASLD but it was lower for lesions smaller than 20 mm. Its specificity was higher for all lesions and those smaller than 20 mm lesions explored by CT. In our study in 1–2 cm nodules, the sensitivity and accuracy are lower in OPTN-UNOS. The main difference on the diagnosis lies on that two additional major findings (washout and capsule appearance) or threshold growth are needed in OPTN-UNOS to diagnose HCC in a 1–2 cm arterial enhancing nodule, while in EASL-EORTC and KLCSG-NCC guideline washout appearance and in LI-RADS washout or threshold growth is sufficient for diagnosis. As of today the current problem, hence main discussion is to increase the accuracy of the diagnosis of 1–2 cm lesions non-invasively. LI-RADS increases its accuracy for these lesions by its latest update.

Incorporation of the radiological guideline of LI-RADS and other clinical guidelines of AASLD and NCCN for radiological evaluation with the recent updates makes these guidelines originated from America closer to each other except OPTN-UNOS. The expedited update of LI-RADS especially for 1–2 cm lesions distinguishes the similarity between LI-RADS and OPTN-UNOS.

There are some limitations in our study. The study was performed with retrospective evaluation and some assumptions needed to be done. The visibility on screening ultrasound is the starting point on some of the guidelines. Ultrasonographic report of all lesions that included in our study could not be reached. Also the requirement of four phase CT could not be fully implemented in our study. Our studies were performed at academic center with high quality and minimum three phase CT studies were included in our study. In our analyses the interval between imaging and pathological assessment is relatively long. But this was unavoidable since many patients received bridging therapies while waiting on transplant list. Such interventions affect the radiological appearance, therefore comparison was made using tests prior to interventions. Prospective studies with more lesions involved, especially between 1–2 cm and <1 cm in diameter should be investigated.

## Conclusions

No statistically significant difference was found between noninvasive diagnostic criteria of HCCs. Overall, LI-RADS had the highest sensitivity and accuracy between the guidelines. OPTN had the highest specificity for cirrhotic livers.

## Supporting information

S1 Table Consensus data. (XLS)
